# Variation in ligamentous laxity in well-functioning total knee arthroplasty is not associated with clinical outcomes or functional ability

**DOI:** 10.1007/s00590-020-02634-1

**Published:** 2020-02-05

**Authors:** David F. Hamilton, Daniel Mandziak, Alexandria Sehgal, Colin R. Howie, Richard Burnett

**Affiliations:** 1 Department of Orthopaedics, University of Edinburgh, 49 Little France Crescent, Edinburgh, EH164SB UK; 2 Department of Orthopaedics, Royal Adelaide Hospital, Adelaide, Australia

**Keywords:** Instability, Revision total knee arthroplasty, Clinical outcomes, Functional assessment

## Abstract

**Background:**

Around 20% of revision knee arthroplasty procedures are carried out for a diagnosis of instability. Clinical evaluation of instability is primarily through physical stress testing of knee ligamentous laxity and joint space opening. It is assumed that increased knee ligament laxity is associated with instability of the knee and, by association, reduced physical function. The range of knee ligament laxity in asymptomatic patients with total knee arthroplasty has however not been reported, nor has the association with measures of physical outcomes.

**Methods:**

Patients who reported being happy with the outcomes of TKA and denied any feelings of knee instability were evaluated at routine follow-up clinicas. Knee ligamentous stability was evaluated seperately by 2 blinded assessors in both coronal and saggital planes. Assessors classified the ligamentous stability as ‘tight’, ‘neutrol’ or ‘loose’. Clinical outcome was evaluated by Oxford Knee Score, patient satisfaction metric, timed performance test, range of motion and lower limb power. Analysis of variance was employed to evaluate variables between groups with post hoc pairwise comparisons.

**Results:**

In total, 42 patients were evaluated. Mean time since index surgery was 46 (SD 8) months. In the coronal plane, 11 (26.2%) were categorised as ‘tight’, 22 (52.4%) as ‘neutral’ and 9 (21.4%) as ‘loose’. In the sagittal plane, 15 (35.7%) were categorised as ‘tight’, 17 (40.5%) as ‘neutral’ and 10 (23.8%) as ‘loose’. There were no between-group differences in outcomes: Oxford Knee Score, range of motion, lower limb power, timed functional assessment score or in satisfaction response in either plane (*p* = 0.05).

**Conclusions:**

We found a range of ligamentous laxity in asymptomatic patients satisfied with the outcome of their knee arthroplasty, and no association between knee laxity and physical ability.

## Introduction

Over 100,000 total knee arthroplasty procedures are carried out in the UK annually [17]. There has been a large increase in surgical volume over time [5], and this trend shows little sign of slowing, with the demand for primary TKA projected to increase by more than 600% by 2030 [14]. Increasing rates of revision total knee replacement are a necessary consequence [11].

Baker et al. [3] showed variation in the clinical outcome of revision total knee replacement dependent on the mode of failure of the primary. Poorer outcomes were reported in those undergoing revision TKA for a diagnosis of unexplained pain or stiffness, compared to those revised for aseptic loosening or osteolysis. A diagnosis of instability accounts for around 20% of revision total knee arthroplasty procedures [2, 19]. While some studies have reported an ability to offer a technical correction and delivery of good outcomes when revising for TKA instability [1, 13], Grayson et al. [8] caution that those revised for flexion instability do not make the same magnitude of improvement in Knee Society Scores or UCLA activity scores compared to those undergoing revision for loosening or infection.

The association between mode of failure and patient outcomes highlights the need to fully understand the underlying mechanism of failure prior to embarking on revision surgery. The causes of total knee replacement instability are multifarious and include inadequate soft-tissue balancing; loss of ligamentous integrity; component wear; improper component sizing and component malpositioning [3, 10]. However, it is often difficult to establish a diagnosis in these cases. In the absence of definitive diagnostic criteria, the clinician’s diagnosis of knee replacement instability is based on patient symptoms, clinical examination and radiological assessment. Patient-reported symptoms can vary from a subtle feeling of instability to frank dislocation. On physical examination, a varus or valgus thrust gait, or hyperextension locking during the stance phase can indicate more severe forms of instability [7] and varus–valgus laxity can be assessed with the knee in 30° flexion [18], with a view to evaluating flexion instability. On that basis, flexion instability is commonly accompanied by collateral ligament laxity [4]. Physical assessment is primarily through manual stress testing of knee ligamentous laxity and evaluation of joint space opening. It is assumed that the increased ligament laxity is associated with knee instability and by association restricted physical function.

We are not aware of any data for the variation of ligamentous laxity in well-functioning total knee arthroplasties nor the relationship between joint laxity and physical ability following TKA. These data are essential to contextualise any ‘pathological laxity’ findings in patients with instability symptoms. Our aim was to evaluate the relationship between clinical examination of ligamentous laxity and functional parameters in patients with a good outcome following TKA.

## Patients and methods

Ethical approval was gained from the local research ethics committee (ref. 11/AL/0079). We prospectively assessed a series of consecutive patients returning to the study centre for routine review of a primary total knee arthroplasty as part of a long-term follow-up project who expressed that they were satisfied with their outcome. Review took place at clinics where the assessors were all present over a 3-month period. All patients had routine primary total knee replacement for a diagnosis of osteoarthritis performed at the study centre. Surgery was carried out by multiple consultant orthopaedic surgeons and their supervised trainees. As is routine surgical practice at our centre, cruciate retaining implants were used in all cases, and the patella was not resurfaced. All patients received identical post-operative care in accordance with the standard protocol of our unit. Rehabilitation included mobilisation on the day of surgery and inpatient physiotherapy.

### Clinical assessment

Knee ‘laxity’ was evaluated through clinical physical examination in both coronal and sagittal planes: medial/lateral stress testing of the collateral ligaments at 30° knee flexion and anterior drawer test of the posterior cruciate ligament at 90° flexion as is recommended as flexion instability evaluations [18], separately by 2 trained assessors (DFH and DM) who were blind to the patient’s opinion of their knee stability and each other’s evaluation. Assessors graded the knee as ‘tight’ (no joint opening), ‘neutral’ (5 mm of joint opening) or ‘loose’ (10 mm of joint opening). Assessors agreed on clinical classification in 93% of cases, In the 3 cases where opinion differed, a third assessor (RB) reviewed the patient blind to the other opinions and consensus was reached.

### Radiographic assessment

Post-operative antero-posterior and lateral radiographs were evaluated to establish coronal and sagittal component alignment to control for any confounding influence of implant positioning on ligamentous laxity. Femoral and tibial component alignment was evaluated on the coronal film, measured relative to the femoral/tibial shaft, where 90° represents implant alignment perpendicular to the shaft axis. Femoral component flexion angle and tibial slope were evaluated relative to the axis of the femoral/tibial shaft on the lateral film, where 90° represents 0° flexion/slope.

### Outcome assessments

Patient-reported outcome was measured with the Oxford Knee Score (OKS), a frequently used and well-validated 12-item response questionnaire designed to assess the patient’s perceived pain and functional ability [6]. Scores range from 0 to 48 with higher values representing better function. Patient satisfaction scores and feelings of knee instability were recorded using 5-point Likert scales.

Active measures of flexion and extension were determined using universal goniometry, previously demonstrated to achieve a high level of accuracy in the clinical setting and specifically in patients following TKA [12, 21].

The patient’s lower limb power was determined using a Leg Extensor Power Rig (LEP, Nottingham, UK), well validated for use with this population [9, 15]. The LEP consists of a seat and footplate connected through a lever and chain to a flywheel. Application of force accelerates the flywheel from rest, and output is recorded as maximal wattage (*W*) generated. Output was reported as maximal wattage generated in a single leg extension.

The ability to perform daily functional tasks was assessed with the aggregated locomotor function score. This score is a composite timed measure of observed locomotor function using tests of walking, stair ascent/decent, and chair transfers, previously demonstrated to be valid, reliable and responsive [16]. Specifically, patients were asked to walk over a flat eight metre course, ascend and then descend a platform consisting of seven fixed steps, and perform a chair transfer task. Time was recorded using a hand-held stopwatch (Zeon, UK).

### Statistical analysis

Data for parametric variables are reported by means with standard deviations as a measure of dispersion. Data were analysed using Prism Version 7 (Graph Pad Software Inc., CA, USA). Patients were grouped according to ligamentous laxity as determined by clinical examination. Assessor agreement of ‘laxity category’ was excellent (ICC > 0.9). Analysis of variance (ANOVA) was employed to evaluate between-group variations with post hoc pairwise comparisons. Statistical significance was accepted at *p* = 0.05. Analysis was conducted, and is reported, separately for assessments in the coronal and sagittal plane.

## Results

In total, 42 patients were recruited to the study over the 3-month timeline. All patients that were invited to take part in the assessments argreed to do so. All testing was conducted around the time of the patients routine clinic visit, and no further follow-up was necessary. All patients had undergone total knee arthroplasty as a primary procedure for a diagnosis of osteoarthritis. 27 (64%) were female, average age was 73 years (SD 8.1 years), and mean time since surgery was 46.24 months (SD 8.02, range 18–68) (Table 1). No patient reported symptoms of knee instability or of giving way. All were broadly happy with the outcome of surgery. 37 (64%) patients reported being very satisfied with the outcome of surgery, 13 (31%) reported being satisfied, 2 (5%) reported being uncertain, and none reported dissatisfaction.

**Table 1:** Study cohort descriptive statistics

	*N* (%)	Age (years)	Gender (f/m)	Side (l/r)	Implant age (months)
Total cohort	42	73 ± 8.1	27/15	21/21	46.24 ± 8.02
Coronal laxity groups					
Tight	11 (26.2)	71 ± 8.3	7/4	9/2	46.36 ± 3.23
Neutral	22 (52.4)	74 ± 8.1	14/8	6/16	45.27 ± 6.73
Loose	9 (21.4)	74 ± 8.5	6/3	6/3	48.44 ± 13.79
Sagittal laxity groups					
Tight	15 (35.7)	71 ± 8.5	12/3	11/4	42.73 ± 8.76
Neutral	17 (40.5)	72 ± 7.6	8/9	6/11	46.88 ± 3.50
Loose	10 (23.8)	76 ± 7.8	7/3	4/6	50.40 ± 10.56

### Coronal plane

Of the 42 patients, 11 (26.2%) were categorised as ‘tight’, 22 (52.4%) as ‘neutral’ and 9 (21.4%) as ‘loose’. There were no between-group differences in age (*p* = 0.63), gender (*p* = 0.22), operated side (*p* = 0.49) or time since index surgery (*p* = 0.33).

There were no differences between groups in radiographic parameters (femoral coronal alignment *p* = 0.52, femoral flexion angle *p* = 0.52, tibial coronal alignment *p* = 0.77, tibial slope *p* = 0.31) (Table 2).

**Table 2:** Radiographic evaluation (by coronal/sagittal plane laxity grouping)

Parameter	Tight (°)	Normal (°)	Loose (°)	*p* value
Coronal laxity groups				
Femoral alignment	95.68 ± 1.93	95.05 ± 2.56	95.89 ± 1.50	0.56
Femoral flexion angle	1.68 ± 1.76	1.64 ± 2.32	0.06 ± 3.47	0.24
Tibial alignment	89.18 ± 1.62	88.68 ± 1.73	89.67 ± 2.33	0.39
Tibial slope	86.64 ± 2.72	86.48 ± 2.67	85.06 ± 3.25	0.38
Sagittal laxity groups				
Femoral alignment	94.97 ± 2.83	95.95 ± 1.72	95.25 ± 1.92	0.52
Femoral flexion angle	1.97 ± 1.83	1.00 ± 3.14	0.85 ± 2.2	0.52
Tibial alignment	89.30 ± 1.37	88.8 ± 2.04	88.85 ± 2.22	0.78
Tibial slope	86.03 ± 2.54	86.94 ± 2.69	85.25 ± 3.33	0.31

**Fig. 1:**
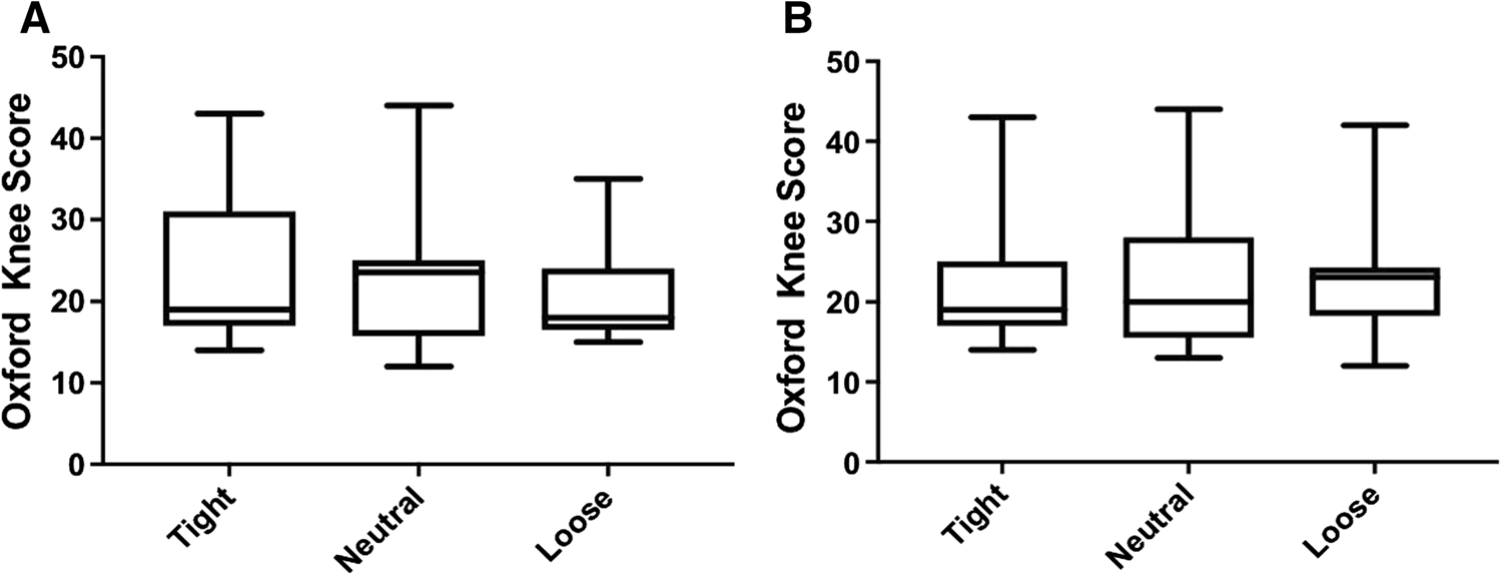
Oxford Knee Scores by laxity classification (**a** coronal plane clinical assessment, **b** sagittal plane clinical assessment)

**Fig. 2:**
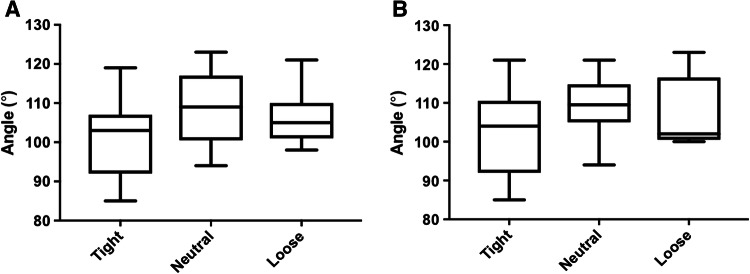
Knee range of motion by laxity classification (**a** coronal plane clinical assessment, **b** sagittal plane clinical assessment)

**Fig. 3:**
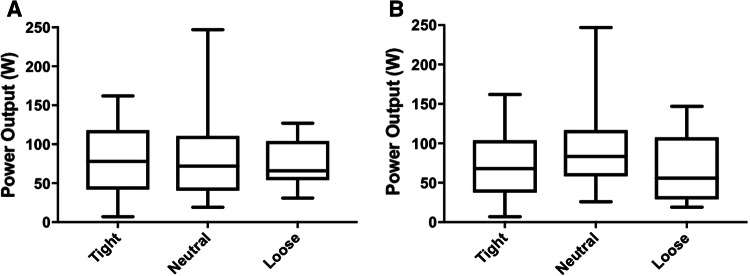
Power output by laxity classification (**a** coronal plane ligament assessment, **b** sagittal plane ligament assessment)

**Fig. 4:**
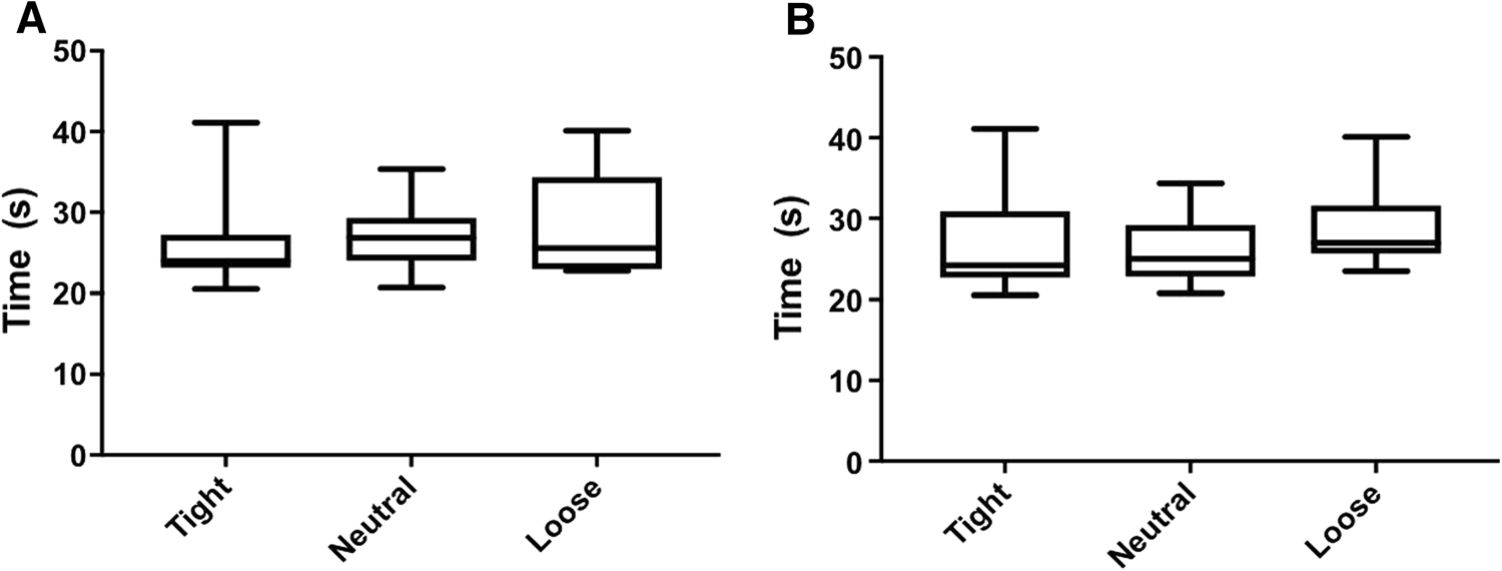
Time functional performance by laxity classification (**a** coronal plane clinical assessment, **b** sagittal plane clinical assessment)

There were no between-group differences in outcomes: Oxford Knee Score (*p* = 0.95, Fig. 1a), range of motion (*p* = 0.33, Fig. 2a), lower limb power output (*p* = 0.23, Fig. 3a), timed functional assessment score (*p* = 0.29, Fig. 4a), or in satisfaction response (*p* = 0.49). There were no significant post hoc pairwise interactions for any evaluation.

### Sagittal plane

Of the 42 patients, 15 (35.7%) were categorised as ‘tight’, 17 (40.5%) as ‘neutral’ and 10 (23.8%) as ‘loose’. There were no between-group differences in age (*p* = 0.18), gender (*p* = 0.70), operated side (*p* = 0.65) or time since index surgery (*p* = 0.33) (Table 1).

There were no differences between groups in radiographic parameters (femoral coronal alignment *p* = 0.56, femoral flexion angle *p* = 0.24, tibial coronal alignment p-0.40, tibial slope *p* = 0.38) (Table 2).

There were no between-group differences in outcomes: Oxford Knee Score (*p* = 0.78, Fig. 1b), range of motion (*p* = 0.13, Fig. 2b), lower limb power output (*p* = 0.98, Fig. 3b), timed functional assessment score (*p* = 0.55, Fig. 4b), or in satisfaction response (*p* = 0.80). There were no significant post hoc pairwise interactions for any evaluation.

## Discussion

This study highlights the variation in joint laxity apparent on physical examination following successful TKA, and the limitations of a static clinical evaluation in terms of defining knee replacement ‘stability’. In this cohort of asymptomatic satisfied patients, nobody reported a feeling of knee instability yet a range of laxity and joint space opening was evident in both planes of reference. There were no differences in radiographic positioning of the implant, patient demographics, patient-reported outcome score, range of motion, ability to accomplish timed functional tasks, lower limb power or satisfaction with outcomes between the 3 clinically determined laxity classifications.

Many surgeons remain somewhat sceptical as to the diagnosis of instability, feeling it poorly defined and something of a get-out-of-jail diagnosis for the unhappy knee replacement. Vince et al. [20] suggest that the patient’s report of instability is not a diagnosis but a presenting complaint, and that clinical examination is the key factor in determining the correct course of action. Interestingly, had the patients we assessed as having ‘loose’ knee on physical examination also reported symptoms of flexion instability, that combination of subjective and objective factors could quite reasonably have been sufficient diagnostic criteria to consider revision surgery. This of course is a speculative situation. Revision surgery is not determined based on isolated clinical findings, but these can be instrumental in determining a view as to the underlying issues. Unless the patients’ symptoms are specific to flexion instability, the presence of ligamentous laxity may not correlate with the problem, and the assessment may in fact risk confirmation bias. As such, we highlight the importance of the subjective evaluation and extended clinical work-up of such cases. Flexion instability is generally considered an early failure modality—with revision surgery typically occurring in the first 4 years following the index procedure [20].

The ability of a patient to tolerate the degree of mechanical laxity in the knee may influence their feeling of laxity and reporting of symptomology. We speculate that some patients may be able to compensate functionally for ‘primary’ (ligamentous) instability through ‘secondary’ (muscle) stabilisation. That the patients we defined as ‘loose’ were able to generate the same muscle power as the others supports this concept of coping with ligamentous laxity using secondary restraints and suggests an important avenue for further research. In particular, it may be that there is an association between the ability of the patient to accommodate excess knee motion using muscle stabilisation and time to onset of instability symptomology, allowing for the presentation of ‘late primary instability’ where the initial ability to accommodate the laxity has been dissipated through time and perhaps progressive physical dysfunction associated with age. If some patients can tolerate a degree of instability, there may be a role for physical therapy to optimise or maintain muscle function as in some sports injuries in those for whom revision surgery carries substantial risks.

## Strengths and limitations

We undertook an evaluation of seemingly well-functioning implants in satisfied patients returning for a routine review to evaluate the variation in ligamentous laxity and relationship between this and measures of physical function and clinical outcome. As such, we cannot comment directly as to the clinical presentation of the unstable total knee replacement but apply our data for context. Comparative evaluation of ligamentous laxity in those presenting with instability symptoms would be an interesting avenue for future research. It is important to note that we present data specific to ligamentous laxity in the flexed knee. We cannot comment on extension laxity or indeed global ligamentous laxity. It is likely this is a different group entirely. A further limitation is the post-operative recruitment process results in a lack of details as to the presentation prior to surgery. According to clinical notes, all patients were ‘routine’ primary TKA for a diagnosis of osteoarthritis, but we cannot comment on their inherent ligamentous laxity prior to surgery. We also assume that laxity presentation has been consistent since the time of surgery. There were no comments on the operative notes that suggested the surgeon had failed to properly balance the knee intra-operatively, suggesting resultant ligament balance to be acceptable at the time of surgery. A future, longitudinal study design would perhaps be useful to comment on this.

The clinical physical evaluation is by nature somewhat subjective and dependent on the clinician’s skill and experience. Despite being based on the ‘clinical feel’, joint space opening is typically reported in millimetres. The ability to accurately and reliably report millimetres of joint opening is challenging; thus, we opted to report a categorical variable (tight, neutral or loose) to represent our evaluated magnitude of opening. We believe this assessment is reflective of the reality of clinical practice; however we accept that we are perhaps reporting our impression of joint opening in descriptive terms as opposed to the actual millimetres of joint opening we think we have evaluated. We suggest this is a reproducible methodology allowing consistency amongst assessors and did result in a high consistency in blind assessor report (ICC > 0.9), but this may be related to the consistency of training and perception of the individual assessors. As such, wider consistency of laxity report cannot be assumed across varied settings. It should be emphasised that the labels of ‘tight’, ‘neutral’ and ‘loose’ we apply are not a judgement on the ‘correct’ laxity that the patient ‘should’ present with. Clearly, there is a range of acceptable ligamentous laxity in patients following TKA. Indeed, some may prefer ‘tight’ or ‘loose’ TKAs. The lack of association between laxity and outcomes may also be complicated by the role of secondary, muscle stabilisation accommodating knee laxity. We think this concept is attractive, but accept that it cannot be established with these data; thus, we propose this as an avenue for further research.

In conclusion, we found a range of ligamentous laxity in satisfied patients following total knee arthroplasty and no association with physical ability. That a large proportion of patients were lax, but with no instability symptoms, suggests potential coping mechanisms that facilitate dynamic function. Caution is advised when a patient with non-specific joint problems following TKA presents with clinically detected knee laxity. Unless symptoms match the clinical examination, a finding of instability may be a variation of normal. This may partly explain the poor results of revision for instability noted by others.
